# Adipocytes reprogram prostate cancer stem cell machinery

**DOI:** 10.1007/s12079-023-00738-x

**Published:** 2023-03-20

**Authors:** Fabrizio Fontana, Martina Anselmi, Patrizia Limonta

**Affiliations:** Department of Pharmacological and Biomolecular Sciences, Università degli Studi di Milano, Milan, Italy

**Keywords:** Prostate cancer, Obesity, Adipocytes, Cancer stem cells, EMT, Metastasis, Chemoresistance

## Abstract

It is now well-established that an obese condition correlates with a higher risk of prostate cancer (PCa). A crosstalk between adipose tissue and PCa has been observed but is still poorly characterized. Herein, we demonstrated that 3T3-L1 adipocyte conditioned media (CM) could endow PC3 and DU145 PCa cells with stemness properties, by stimulating their sphere formation ability and promoting CD133 and CD44 expression. Moreover, after exposure to adipocyte CM both PCa cell lines underwent partial epithelial-to-mesenchymal transition (EMT), with E-/N-cadherin switch and Snail upregulation. Specifically, these changes in PC3 and DU145 cell phenotype were accompanied by increased tumor clonogenic activity and survival, as well as by enhanced invasion, anoikis resistance and matrix metalloproteinase (MMP) production. Finally, adipocyte CM-treated PCa cells exhibited reduced responsiveness to both docetaxel and cabazitaxel, demonstrating greater chemoresistance. Overall, these data indicate that adipose tissue can effectively contribute to PCa aggressiveness by reprogramming the cancer stem cell (CSC) machinery.

**Graphical abstract:**

Adipocytes endow prostate cancer cells with stem-like properties and mesenchymal traits, increasing their tumorigenicity, invasion and chemoresistance.
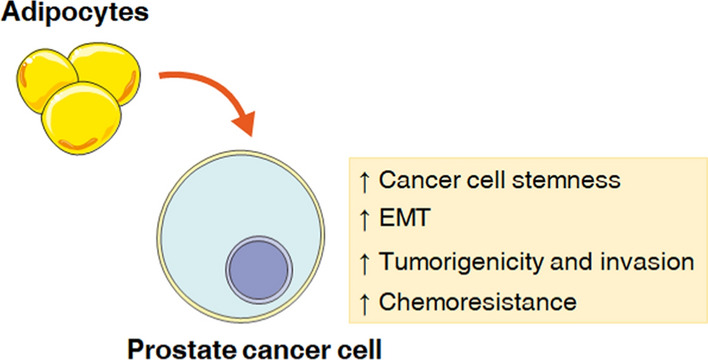

## Introduction

Prostate cancer (PCa) is usually an indolent malignancy with multiple therapeutic options. Active surveillance is still the preferred approach for men with less-aggressive tumors, and localized carcinomas can be successfully managed by surgery, radiotherapy or androgen deprivation treatment (Horwitz and Hanks 2000; Perlmutter and Lepor 2007; Lawrentschuk et al. 2010). However, about 40% of patients experience disease recurrence, and the tumor progresses towards a condition of castration resistance, characterized by high aggressiveness, increased metastatic potential and poor prognosis (Fontana et al. 2020; Fontana and Limonta 2021). Therefore, a deeper understanding of the mechanisms responsible for PCa evolution is urgently needed, with many studies currently focusing on the identification of specific molecular signatures that might be targeted by ad hoc therapies (Fontana et al. 2022b).

Several risk factors have already been identified for PCa. These include elder age, race/ethnicity and family history (Leitzmann and Rohrmann 2012). Acquired or inherited genetic alterations in tumor suppressor genes and oncogenes have also been associated with the risk of developing this carcinoma (Fontana et al. 2022b). On the other hand, among the numerous modifiable factors involved in PCa initiation or progression, obesity is one of the most extensively studied.

In the last years, obesity has reached epidemic proportions, with almost two billion of adults being overweight and 600 million of them being obese worldwide (Chooi et al. 2019). This condition results from a complex interaction of genetic predisposition and the environment, with increased caloric intake and decreased physical activity leading to chronic energy imbalance and expansion of dysfunctional adipose mass (Hruby et al. 2016; Lin and Li 2021). Although the existence of a specific link between excessive overweight and PCa insurgence is still controversial, obesity has been widely associated with a more aggressive tumor and limited therapeutic outcomes (Freedland and Aronson 2004; Cao and Giovannucci 2016; Ferro et al. 2017; Nassar et al. 2018; Estève et al. 2020). Several mechanisms have been proposed to explain this correlation, including hyperinsulinemia, adipokine overproduction and increased hormone synthesis (Freedland and Aronson 2004; Cao and Giovannucci 2016; Ferro et al. 2017; Nassar et al. 2018; Estève et al. 2020). However, data about the specific phenotypic changes and signaling pathways responsible for the obesity-driven PCa evolution are still scanty, with just a few studies reporting the involvement of some pro-tumor proteins, such as HIF, JNK and JAK/STAT cascade, in the adipose-to-tumor cell communication (Dumas and Brisson 2021). In particular, the effects of adipocytes on the cancer stem cell (CSC) machinery, the cellular compartment responsible for tumor metastasis and chemotherapy escape (Skvortsov et al. 2018), have not been investigated yet.

In the present study, we further dissected the biological mechanisms underlying the crosstalk between adipose tissue and PCa, with a focus on the cellular and molecular alterations implicated in the tumor switch towards an aggressive phenotype.

## Material and methods

### Chemicals

Docetaxel and cabazitaxel were from Sigma-Aldrich (Milano, Italy).

The primary antibodies CD133 (64,326), CD44 (3570), MMP2 (4022), MMP9 (3852), E-cadherin (3195), N-cadherin (13,116), Snail (3879) were from Cell Signaling Technology Inc. (Danvers, MA, USA). α-Tubulin (T6199) was from Sigma-Aldrich. All the antibodies were used at the concentration 1:1000.

Horseradish-peroxidase-conjugated secondary antibody and enhanced chemiluminescence reagents were from Cyanagen (Bologna, Italy).

### Cell lines and cell culture

PC3 and DU145 PCa cells were from American Type Culture Collection (ATCC, Manassas, VA, USA), and they were cultured in RPMI media supplemented with 7.5% and 5% FBS respectively, glutamine and antibiotics, in humidified atmosphere of 5% CO_2_/95% air at 37 °C. 3T3-L1 pre-adipocytes were also from ATCC and were grown in DMEM media supplemented with 10% FBS, glutamine and antibiotics. Original stocks of cells were stored frozen in liquid nitrogen. After resuscitation, cells were kept in culture for no more than 10–12 weeks. They were detached through trypsin–EDTA solution and passaged once/week.

### 3T3-L1 cell differentiation and conditioned media collection

3T3‐L1 pre-adipocyte differentiation was initiated by replacing regular media with induction media containing 10% FBS and 500 µM 3‐isobutyl‐1‐methylxanthine, 1 µM dexamethasone, 1 µg/ml insulin and 1 µM rosiglitazone. After 3 days, the induction media was replaced with DMEM containing 10% FBS and 1 µg/ml insulin, and cells were left growing for other 4 days. After that, mature adipocytes were maintained in regular media (DMEM with 10% FBS) for additional 3 days. Finally, media change with serum-free DMEM was made: after 24 h, conditioned media (CM) was collected and stored at − 80 °C. Serum-free DMEM was used as corresponding control.

### Prostasphere formation assay

PCa cells were pre-treated with adipocyte CM for 24 h and then plated in low attachment 25-cm^2^ flasks (5 × 10^5^ cell/flask) and incubated with serum-free DMEM-F12 containing 10 ng/ml recombinant human fibroblast growth factor, 10 ng/ml recombinant human epidermal growth factor, 4 µg/ml insulin and 0.2% B27 for 7 days. Prostaspheres were counted by using a Zeiss Axiovert 200 microscope.

### Colony formation assay

PCa cells were seeded (100–250 cells/well, depending on the cell type) in 6-well plates. After 48 h, cells were treated with adipocyte CM for 72 h and then cultured for 7–10 days in complete RPMI. Colonies were fixed with 70% methanol and stained with Crystal Violet 0.15%.

### Annexin V/PI apoptosis assay

PCa cells were plated (1.5 × 10^5^ cells/dish) in 6‐cm dishes. After 48 h, they were treated with adipocyte CM for 96 h. Cells were then harvested, washed in PBS and incubated with Annexin V and PI, using the eBioscience™ Annexin V-FITC Apoptosis Detection Kit. The flow cytometry analyses were performed with a Novocyte3000 instrument (ACEA Biosciences, San Diego, CA, USA). Data were analyzed with Novoexpress software.

### Morphological analysis

PCa cells were seeded (5 × 10^4^) in 6-well plates and cultured in adipocyte CM for 96 h. Their morphology was then analyzed by light microscopy. A Zeiss Axiovert 200 microscope was used for the analysis, and cells were observed with a 20 × 1.4 objective lens connected to a Coolsnap Es CCD camera (Roper Scientific-Crisel Instruments, Roma, Italy).

### Boyden chamber assay

PCa cells were plated (1.5 × 10^5^ cells/dish) in 6‐cm dishes. After 48 h, they were treated with adipocyte CM for 24 h. Cells were then harvested, resuspended in serum-free culture media and placed in the open-bottom wells of the upper compartment of the Boyden chamber. The chemoattractant (FBS 5%) was placed in the lower compartment of the chamber. The two compartments were separated by polyvinylpyrrolidone-free polycarbonate porous membrane (8-µm pores) precoated with gelatin (0.2 mg/ml in PBS). The chamber was then kept in the incubator for 6 h. After that, invaded cells on the lower surface of the membranes were fixed, stained with Diff-Quick staining kit (DADE, Dudingen, Switzerland) and counted in three randomly selected microscope fields.

### Anoikis assay

PCa cells were seeded (5 × 10^4^) in ultra-low attachment 24-well plates and cultured in adipocyte CM for 48 h. Cells were then harvested, stained with Trypan blue 0.4% (1:1 v/v) and counted by Luna automated cell counter (Logos Biosystems, Annandale, VA, USA).

### MTT viability assay

Cells were seeded at a density of 3 × 10^4^ cells/well in 24‐well plates. After 48 h, they were pre-treated with adipocyte CM for 24 h and then exposed to docetaxel (10 nM) or cabazitaxel (5 nM) for 48 h. The medium was then changed with MTT solution (0.5 mg/mL) in RPMI without phenol red and FBS; cells were incubated at 37 °C for 30 min and violet precipitate was dissolved with isopropanol. Absorbance at 550 nm was measured through an EnSpire Multimode Plate reader (PerkinElmer, Milano, Italy).

### Western blot analysis

Cells were seeded at 5 × 10^5^ cells/dish in 10‐cm dishes. After each treatment, they were lysed in RIPA buffer; protein preparations (25 µg) were resolved on SDS‐PAGE and transferred to nitrocellulose membranes. Membranes were incubated with the specific primary antibodies. Detection was done using horseradish peroxidase‐conjugated secondary antibodies and enhanced chemiluminescence (Westar Etac Ultra 2.0, XLS075,0100, Cyanagen). α-Tubulin was utilized as a loading control.

### Statistical analysis

Statistical analysis was performed with a statistic package (GraphPad Prism5, GraphPad Software San Diego, CA, USA). Data are represented as the mean ± SEM of three independent experiments. Differences between groups were assessed by *t*-test or two-way analysis of variance (ANOVA) followed by Bonferroni’s test. A *P* value < 0.05 was considered statistically significant.

## Results

### Adipocytes promote prostate cancer cell stemness

**Fig. 1:**
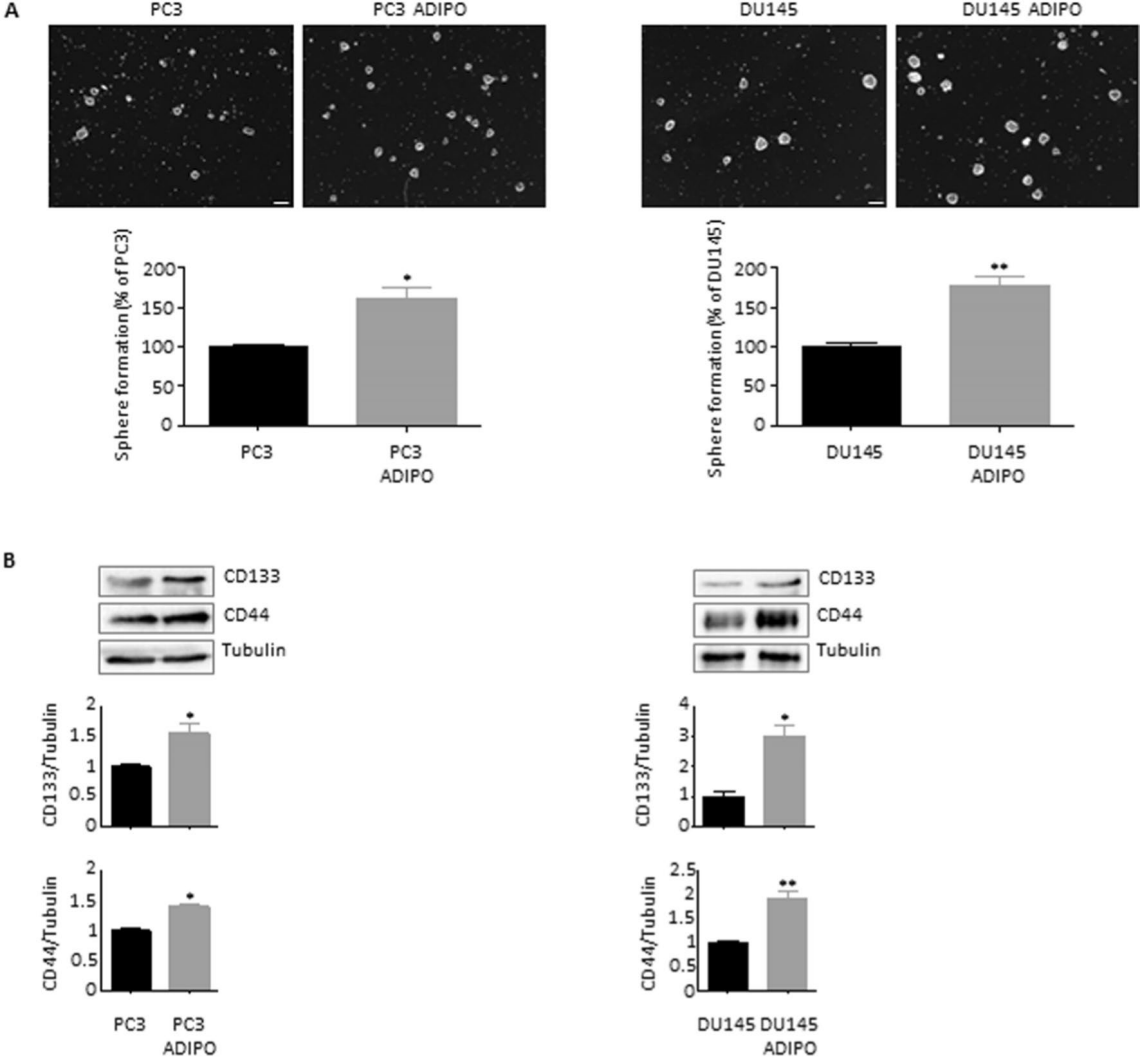
Adipocytes promote prostate cancer cell stemness. (**A**) PC3 and DU145 cells were pre-treated with adipocyte CM for 24 h and then cultured in CSC media for 7 days. Sphere formation assay was performed to determine the spheroidogenic ability of cancer cells. Each experiment was repeated three times. Scale bars are 75 µm. Data represent mean values ± SEM and were analyzed by *t*-test. *P < 0.05 versus PC3 or DU145 (control). **P < 0.01 versus PC3 or DU145 (control). (**B**) Cells were pre-treated with adipocyte CM for 24 h and then cultured in CSC media for 7 days. Western blot analysis was performed to investigate the expression levels of CD133 and CD44 in PC3 and DU145 cells. Tubulin expression was evaluated as a loading control. Data represent mean values ± SEM and were analyzed by *t*-test. *P < 0.05 versus PC3 or DU145 (control). **P < 0.01 versus PC3 or DU145 (control)

To decipher the influence of adipose tissue on tumor cells, 3T3‐L1 preadipocytes were first differentiated into adipocytes. Mature adipose cells were then incubated in serum-free media; after 24 h, CM was collected, and its effects were tested on PC3 and DU145 PCa cell lines. As evidenced in Fig. 1A, 3T3-L1 adipocyte media significantly promoted tumor cell spheroidogenic ability, a typical feature of CSCs. In addition, an enrichment in stemness markers, namely CD133 and CD44, was observed (Fig. 1B). Thereby, these data point to a key role of adipose tissue in determining the insurgence of a stem-like state in PCa.

### Adipocytes stimulate prostate cancer cell tumorigenicity and survival

**Fig. 2:**
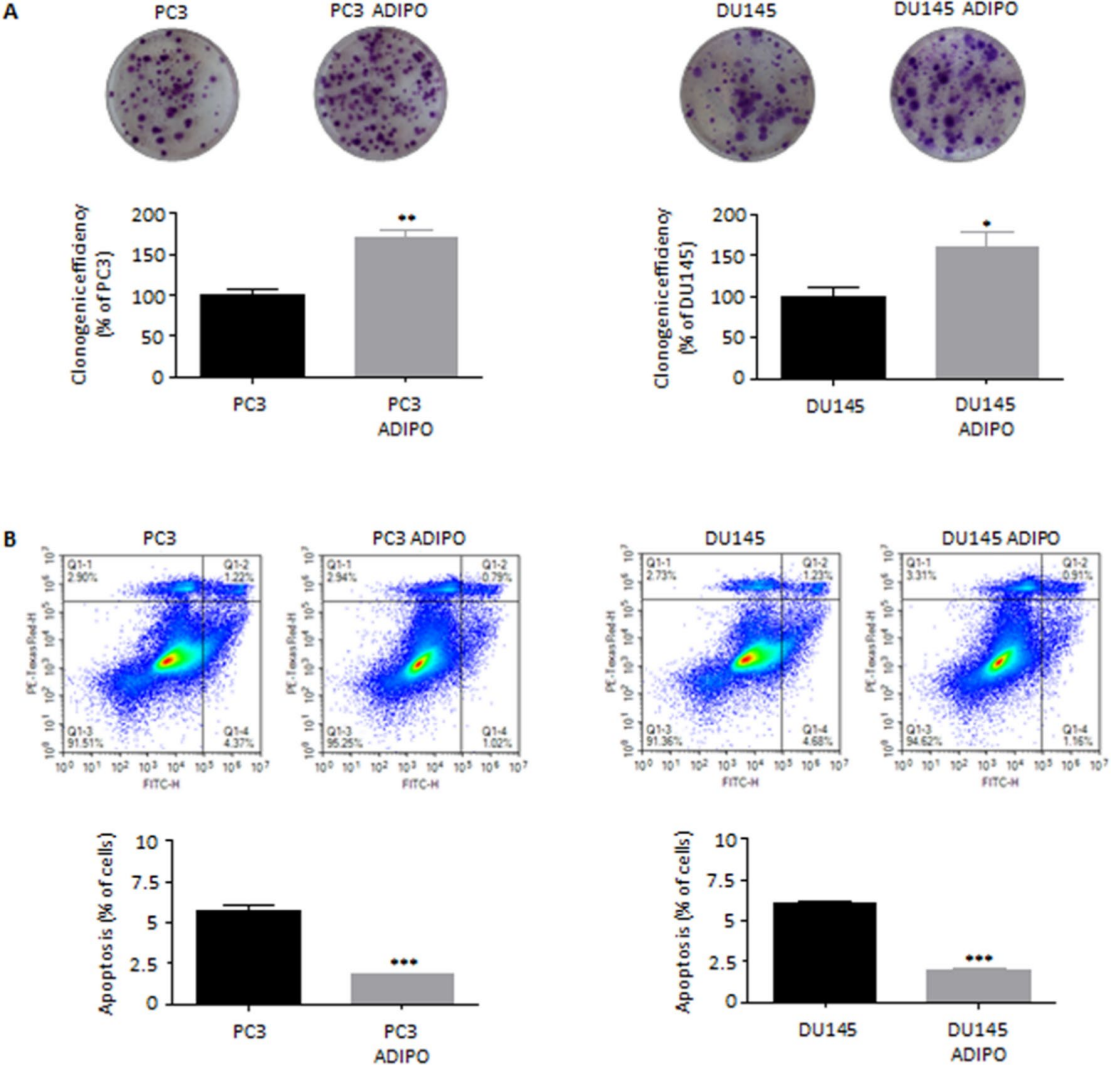
Adipocytes stimulate prostate cancer cell tumorigenicity and survival. (**A**) PC3 and DU145 cells were pre-treated with adipocyte CM for 72 h and then cultured in proper media for 7–10 days. Colony formation assay was performed to determine the clonogenic activity of cancer cells. Each experiment was repeated three times. Data represent mean values ± SEM and were analyzed by *t*-test. *P < 0.05 versus PC3 or DU145 (control). **P < 0.01 versus PC3 or DU145 (control). (**B**) Cells were incubated in adipocyte CM for 96 h. Apoptotic rates were then evaluated by cytofluorimetric analysis after staining with eBioscience™ Annexin V-FITC Apoptosis Detection Kit (according to the manufacturer’s protocol). Each experiment was repeated three times. Data represent mean values ± SEM and were analyzed by *t*-test. ***P < 0.001 versus PC3 or DU145 (control)

CSCs are known to display specific self-renewal and tumorigenic properties (Marzagalli et al. 2021). In accordance with this notion, exposure of PC3 and DU145 cells to adipocyte CM was followed by an increase in their clonogenic activity (Fig. 2A). Similarly, the number of living cells was higher in the conditioned groups compared to controls, with just a 2% rate of spontaneous apoptosis (Fig. 2B). Collectively, these results demonstrate the involvement of adipose tissue in PCa cell tumorigenicity and survival.

### Adipocytes induce partial EMT in prostate cancer cells

**Fig. 3:**
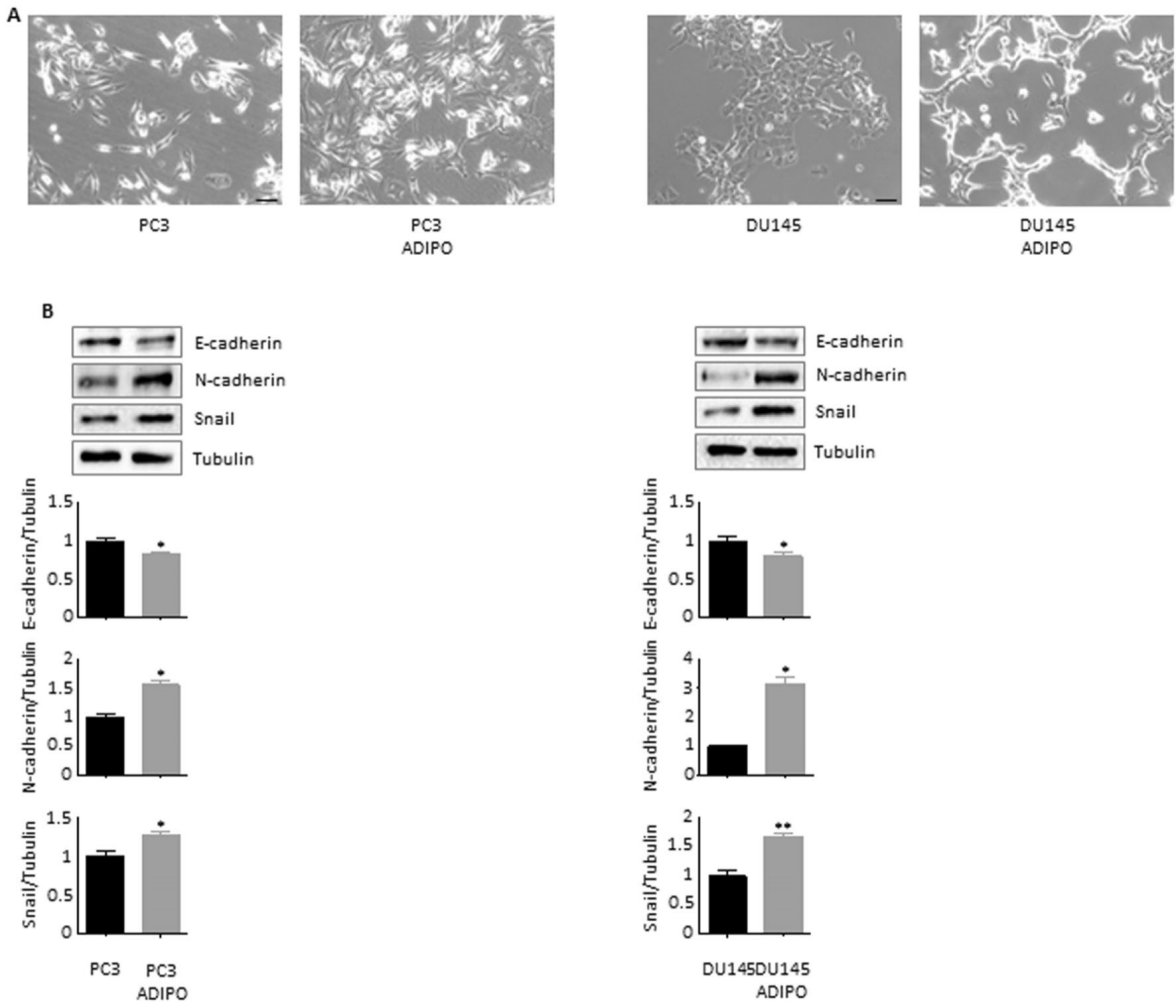
Adipocytes endow prostate cancer cells with mesenchymal properties. (**A**) PC3 and DU145 cells were incubated in adipocyte CM for 96 h. Cell morphology was then evaluated by light microscopy. Each experiment was repeated three times. Scale bars are 40 µm. (**B**) After adipocyte CM treatment (24 h), Western blot analysis was performed to investigate the expression levels of E-cadherin, N-cadherin and Snail in PC3 and DU145 cells. Tubulin expression was evaluated as a loading control. Data represent mean values ± SEM and were analyzed by *t*-test. *P < 0.05 versus PC3 or DU145 (control). **P < 0.01 versus PC3 or DU145 (control)

PCa evolution has been widely associated with the emergence of an aggressive phenotype, characterized not only by the activation of stem-related programs but also by improved cellular plasticity (Skvortsov et al. 2018). In particular, partial or total EMT has been shown to drive PCa metastasis and therapy escape (Montanari et al. 2017). Intriguingly, treatment of PC3 and DU145 cell lines with adipocyte CM led to the acquisition of an elongated and spindle-shaped morphology, a typical mesenchymal-like characteristic (Odero-Marah et al. 2018) (Fig. 3A). Molecularly, conditioned cells displayed E-cadherin downregulation and N-cadherin overexpression, indicating a shift towards an intermediate EMT state (Odero-Marah et al. 2018); in support of this observation, an increase in the levels of Snail, the master regulator of E-/N-switch during PCa dedifferentiation (Smith and Odero-Marah 2012), was also found (Fig. 3B). Of note, the induction of these pro-tumor cascades generally correlates with the acquisition of the above CSC traits (Skvortsov et al. 2018).

### Adipocytes drive prostate cancer cell invasion

**Fig. 4:**
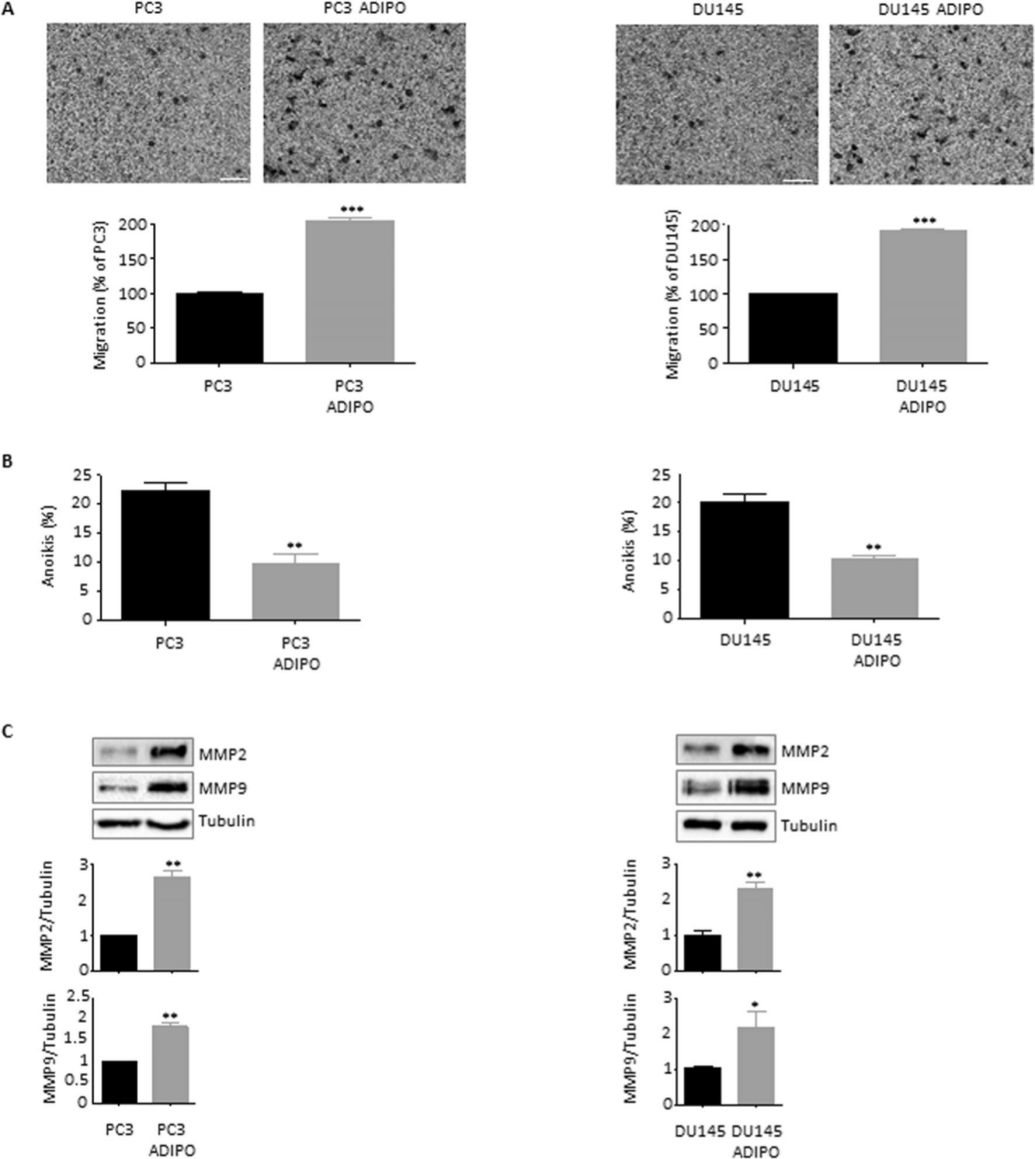
Adipocytes drive prostate cancer cell invasion. (**A**) PC3 and DU145 cells were incubated in adipocyte CM for 24 h. Cell invasion was then evaluated by Boyden chamber assay. Each experiment was repeated three times. Scale bars are 100 µm. Data represent mean values ± SEM and were analyzed by *t*-test. ***P < 0.001 versus PC3 or DU145 (control). (**B**) Cells were incubated in adipocyte CM for 48 h. Anoikis was then evaluated by Trypan blue exclusion assay. Each experiment was repeated three times. Data represent mean values ± SEM and were analyzed by *t*-test. **P < 0.01 versus PC3 or DU145 (control). (**C**) After adipocyte CM treatment (24 h), Western blot analysis was performed to investigate the expression levels of MMP2 and MMP9 in PC3 and DU145 cells. Tubulin expression was evaluated as a loading control. Data represent mean values ± SEM and were analyzed by *t*-test. *P < 0.05 versus PC3 or DU145 (control). **P < 0.01 versus PC3 or DU145 (control)

Given the strict link existing between cancer stemness, EMT and tumor spread (Fontana et al. 2019; Bakir et al. 2020), changes in the metastatic traits of adipocyte CM-treated cancer cells were analyzed. Figure 4A highlights the ability of 3T3-L1 mature adipose cells to stimulate the invasion of both PC3 and DU145 cell lines. More importantly, conditioned PCa cells were found to successfully escape from anoikis, an anchorage-dependent form of cell death induced by lack of correct cell/extracellular matrix adhesion (Paoli et al. 2013) (Fig. 4B). As expected, this was paralleled by the activation of metastasis-sustaining matrix metalloproteinase 2 and 9 (MMP2 and 9) (Fig. 4C). Once again, these findings corroborate the hypothesis that adipose mass can fuel PCa metastatic stem cell machinery.

### Adipocytes confer chemoresistance to prostate cancer cells

**Fig. 5:**
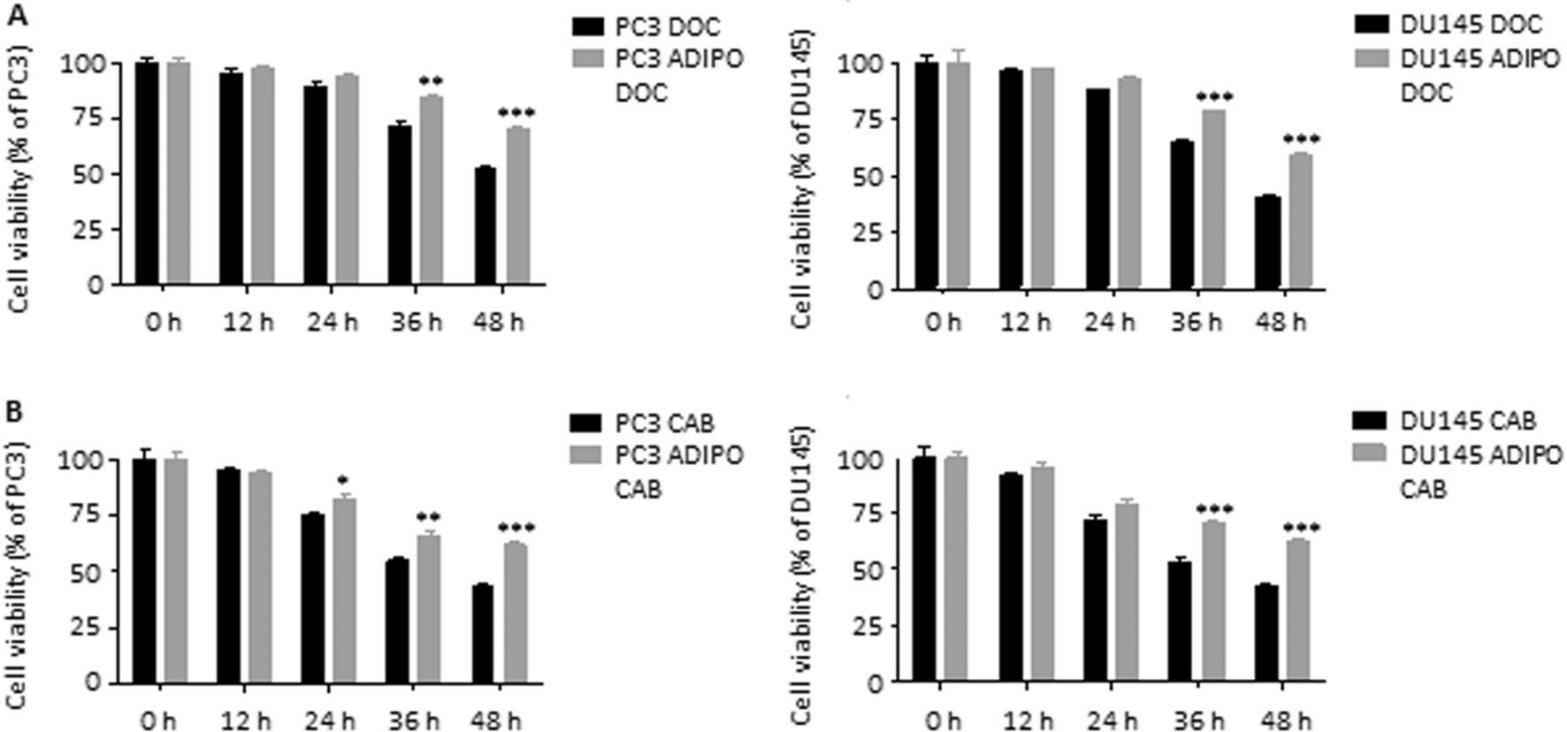
Adipocytes confer chemoresistance to prostate cancer cells. (**A**) PC3 and DU145 cells were incubated in adipocyte CM for 24 h and then treated with docetaxel (10 nM) for 48 h. Cell viability was then evaluated by MTT assay. Each experiment was repeated three times. Data represent mean values ± SEM and were analyzed by two-way ANOVA followed by Bonferroni's test. **P < 0.01 versus PC3 DOC or DU145 DOC (control). ***P < 0.001 versus PC3 DOC or DU145 DOC (control). (**B**) PC3 and DU145 cells were incubated in adipocyte CM for 24 h and then treated with cabazitaxel (5 nM) for 48 h. Cell viability was then evaluated by MTT assay. Each experiment was repeated three times. Data represent mean values ± SEM and were analyzed by two-way ANOVA followed by Bonferroni’s test. *P < 0.05 versus PC3 CAB or DU145 CAB (control). **P < 0.01 versus PC3 CAB or DU145 CAB (control). ***P < 0.001 versus PC3 CAB or DU145 CAB (control)

It is now widely accepted that CSC niches are not only the major drivers of tumor metastasis but also the main modulators of cancer drug resistance. In this regard, PC3 and DU145 cells exposed to adipocyte CM were shown to exhibit reduced sensitivity to both docetaxel and cabazitaxel, two chemotherapeutics commonly used for PCa management (Fig. 5A and B). Overall, this evidence confirms the ability of adipose tissue to directly act on PCa CSC programs, boosting tumor aggressiveness.

## Discussion

Obesity represents a growing health problem, with several studies linking this pathology to lethal PCa. Indeed, obese men have an increased risk of disease progression after radical prostatectomy and are more likely to develop metastases or die from PCa compared to normal weight men (Freedland and Aronson 2004; Cao and Giovannucci 2016; Ferro et al. 2017). Similarly, a high density of periprostatic adipose tissue has been associated with more aggressive tumors (Nassar et al. 2018; Estève et al. 2020). However, the cellular and molecular alterations implicated in the obesity-related PCa progression still need to be elucidated.

In the present study, we investigated the influence of 3T3-L1 adipocyte CM on the phenotype of PC3 and DU145 PCa cell lines, in order to identify the specific signature elicited by adipose tissue in cancer cells.

First, we demonstrated that adipocyte CM could exert a significant pro-tumor activity on PCa cells, by endowing them with CSC properties; specifically, it could promote tumor sphere formation as well as CD133 and CD44 upregulation. Moreover, we observed that 3T3-L1 mature adipose cells could stimulate PCa cell clonogenic activity and resistance to spontaneous apoptosis, increasing overall cellular tumorigenicity and survival. This is in agreement with recent findings evidencing the CSC-enriching effects mediated by adipocytes in various malignancies, including mammary, ovarian and colon carcinoma (Wen et al. 2017; Ladanyi et al. 2018; Goto et al. 2019).

Next, we found that treatment of PCa cells with adipocyte CM led to a partial EMT, characterized by E-/N-cadherin switch and Snail overexpression. These data are consistent with previous reports describing the crucial role of adipose tissue in promoting the development of a plastic phenotype in several tumors, such as breast and pancreatic cancer (Dirat et al. 2011; Wang et al. 2017; Okumura et al. 2017; He et al. 2018). Remarkably, co-activation of CSC and EMT programs has been reported in co-cultures of adipocytes with colorectal carcinoma cells (Wen et al. 2017; Di Franco et al. 2021), further supporting the assumption by which the dialog between adipose mass and tumor cells strongly relies on the stimulation of a complex stem-like signaling network, involving the induction of specific mesenchymal transformation-supporting systems.

To get additional insights into the targets of adipocyte CM, we focused our attention on the analysis of PCa cell metastatic potential. Indeed, cancer stemness, EMT and tumor metastasis are commonly recognized as three sides of the same coin (Skvortsov et al. 2018; Marzagalli et al. 2021). On the other hand, extracellular matrix remodeling and cancer spread have been largely observed in numerous tumors following adipose tissue-mediated paracrine stimulation (Dumas and Brisson 2021). In PC3 and DU145 cells, we showed that incubation with adipocyte CM resulted in a markedly enhanced invasion. This was accompanied by anoikis evasion and MMP2 and 9 activation. To our knowledge, this is the first study highlighting a relationship between PCa metastasis and the above phenotypic traits in the context of an obese condition.

Based on the profound correlation occurring between a plastic stem-like state and chemoresistance, we finally validated our conclusions in a pharmacological setting. Indeed, while docetaxel and cabazitaxel have shown great promise in the management of tumor cells per se, they have been little investigated in the context of the heterotypic interactions between cancer cells and adipocytes (Liotti et al. 2021; Fontana et al. 2022a). Here, we reported that treatment of PC3 and DU145 cell lines with adipocyte CM significantly reduced the growth-suppressing effects of both taxanes: on one side, this evidence confirms the ability of adipose mass to modulate tumor response to therapy, as previously highlighted in breast, liver and ovarian cancer, leukemia and melanoma (Dumas and Brisson 2021); on the other side, it points to the key role of CSC plasticity in the obesity-mediated PCa evolution.

## Conclusions

In summary, these results provide a deeper understanding of the biological mechanisms underlying the interactions between adipose tissue and PCa, demonstrating that the first can reprogram the CSC machinery to promote tumor progression.
